# Chemical and environmental vector control as a contribution to the elimination of visceral leishmaniasis on the Indian subcontinent: cluster randomized controlled trials in Bangladesh, India and Nepal

**DOI:** 10.1186/1741-7015-7-54

**Published:** 2009-10-05

**Authors:** Anand B Joshi, Murari L Das, Shireen Akhter, Rajib Chowdhury, Dinesh Mondal, Vijay Kumar, Pradeep Das, Axel Kroeger, Marleen Boelaert, Max Petzold

**Affiliations:** 1 Institute of Medicine, Tribhuvan University, Kathmandu, Nepal; 2 BP Koirala Institute of Health Sciences, Dharan, Nepal; 3 NIPSOM, Mohakhali, Dhaka, Bangladesh; 4 ICDDRB, Dhaka, Bangladesh; 5 RMRIMS (ICMR) Patna-800 007, India; 6 Special Programme for Research and Training in Tropical Diseases, World Health Organization, Geneva, Switzerland; 7 Liverpool School of Tropical Medicine, Liverpool, UK; 8 Department of Public Health, Institute of Tropical Medicine, Antwerp, Belgium; 9 Nordic School of Public Health, Göteborg, Sweden; 10 Statistical Research Unit, Göteborg University, Göteborg, Sweden

## Abstract

**Background:**

Bangladesh, India and Nepal are working towards the elimination of visceral leishmaniasis (VL) by 2015. In 2005 the World Health Organization/Training in Tropical Diseases launched an implementation research programme to support integrated vector management for the elimination of VL from Bangladesh, India and Nepal. The programme is conducted in different phases, from proof-of-concept to scaling up intervention. This study was designed in order to evaluate the efficacy of the three different interventions for VL vector management: indoor residual spraying (IRS); long-lasting insecticide treated nets (LLIN); and environmental modification (EVM) through plastering of walls with lime or mud.

**Methods:**

Using a cluster randomized controlled trial we compared three vector control interventions with a control arm in 96 clusters (hamlets or neighbourhoods) in each of the 4 study sites: Bangladesh (one), India (one) and Nepal (two). In each site four villages with high reported VL incidences were included. In each village six clusters and in each cluster five households were randomly selected for sand fly collection on two consecutive nights. Control and intervention clusters were matched with average pre-intervention vector densities.

In each site six clusters were randomly assigned to each of the following interventions: indoor residual spraying (IRS); long-lasting insecticide treated nets (LLIN); environmental management (EVM) or control. All the houses (50-100) in each intervention cluster underwent the intervention measures. A reduction of intra-domestic sand fly densities measured in the study households by overnight US Centres for Disease Prevention and Control light trap captures (that is the number of sand flies per trap per night) was the main outcome measure.

**Results:**

IRS, and to a lesser extent EVM and LLINs, significantly reduced sand fly densities for at least 5 months in the study households irrespective of type of walls or whether or not people shared their house with cattle. IRS was effective in all sites but LLINs were only effective in Bangladesh and India. Mud plastering did not reduce sand fly density (Bangladesh study); lime plastering in India and one Nepali site, resulted in a significant reduction of sand fly density but not in the second Nepali site.

**Conclusion:**

Sand fly control can contribute to the regional VL elimination programme; IRS should be strengthened in India and Nepal but in Bangladesh, where vector control has largely been abandoned during the last decades, the insecticide treatment of existing bed nets (coverage above 90% in VL endemic districts) could bring about an immediate reduction of vector populations; operational research to inform policy makers about the efficacious options for VL vector control and programme performance should be strengthened in the three countries.

## Background

Visceral leishmaniasis (VL, named kala-azar in the Indian sub-continent) is endemic in three countries of South East Asia: Bangladesh, India and Nepal. Approximately 200 million people in the region are at risk and the disease is now being reported in 45 districts in Bangladesh, 52 in India and 12 in Nepal. Of the estimated 500,000 people in the world who are infected each year, nearly 100,000 are estimated to occur in these three countries. VL affects the poorest among the poor in the endemic areas [1].

In 2005 all the three countries agreed to initiate a VL elimination programme with high level political commitment. The target was to reduce the annual VL incidence to 1/10,000 population by 2015. Favouring factors are that: human beings are the only reservoir host; *Phlebotomus argentipes *is the only vector in the region; VL is concentrated in 109 districts in the three countries; the disease is easy to diagnose, even in field settings, through the recently developed rK39 dipstick test; and it can be treated completely with effective drugs [2,3]. The five pillars of the VL elimination strategy are: providing access to early diagnosis and treatment; strengthening disease and vector surveillance; integrated vector management; social mobilization and networking; and operational research [4]. However, there is still a long way to go before we can turn this concept into a reality.

VL was virtually eliminated from the region during the malaria eradication era in the 1950s in response to spraying with dichlorodiphenyltrichloroethane (DDT), but returned after the relaxation of the spraying operations [5]. Today, Bangladesh has largely abandoned vector control operations in favour of VL. In India, however, DDT is still used for blanket indoor residual spraying (IRS). On the other hand, Nepal applies IRS with pyrethroids in a targeted way to those villages in which a case of VL has been reported in the previous year [6].

Providing or promoting the use of long-lasting insecticide treated nets (LLINs) is a potential alternative for sand fly control [7-10] as bed-net use is common practice in Bangladesh [5,9,11] and parts of India and Nepal [8], and the vector, *P. argentipes*, is a night biter in and around houses [12]. Environmental management (EVM) may be another alternative tool for VL vector control. In the rural areas of these countries it is a common practice to plaster the walls and floors of houses and cattle sheds with mud and clay. A sudden drop in the sand fly density was noticed in a pilot study when treated houses were compared with untreated control houses [13].

This study was designed in order to evaluate the efficacy of the different interventions for VL vector management (IRS, LLINs, and EVM) in reducing sand fly density under fairly controlled conditions to better inform the policy makers about the different VL vector control options.

## Methods

### Study design

A cluster randomized controlled trial was used to evaluate the effectiveness of IRS, LLIN and EVM (house wall plastering with mud in Bangladesh or liming in Nepal and India) in the reduction of vector density compared to a control group. Sand fly collections were taken before the application of the interventions and follow-up sand fly collection were taken after 6-months in all the intervention and control areas.

### Study areas

**Figure 1:**
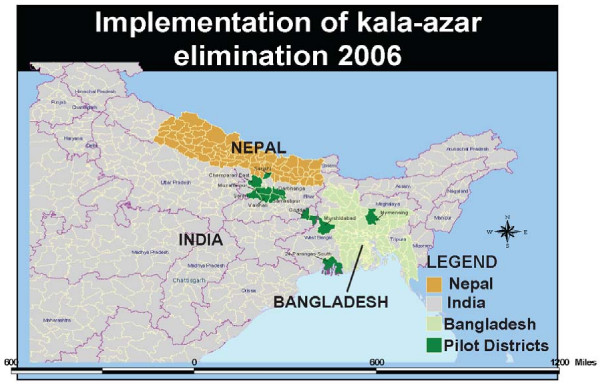
**Map of the participating sites**.

The study sites were selected according to a past reported high VL incidence in the following endemic districts: Fulbaria Upazilla, Mymensingh districts in Bangladesh; Vaishali and Muzzaffarpur districts in India; and Sarlahi, Sunsari and Morang districts in Nepal (Figure 1). Reported cases were based on passive case detection and taken from the Health Information Management System of each of the countries for the past 3 years. The data are comparable for the three endemic countries as they use similar diagnostic criteria based on clinical signs and symptoms and laboratory tests (rk39 rapid tests and parasitological confirmation). Climatic conditions in the study areas are fairly uniform, with a low vector season from December to March due to lower temperatures. Socio-economic conditions (including age structure, the number of people per household and the illiteracy rate) and disease awareness was comparable in each of the study sites. No public or private vector control activities had been undertaken in the study districts during the previous year.

### Sample size

The sample size estimation was based on the vector densities, variation and distribution documented in previous entomological studies and the sand fly reduction in a similar intervention study in Venezuela [14]. Our study assumed that the distribution of sand fly counts follows a negative binomial distribution with a dispersion coefficient of *k *= 0.05 and an intra cluster coefficient (ICC) of 0.03. Further, a reduction from 20 to 5 vectors per trap and per night, and an average of 50 to 100 households per cluster (representing a hamlet or neighborhood), was assumed. The minimum sample size was found to be six clusters per arm, with a total of 24 clusters per study site, in order to achieve the required power of 80% and a significance level of 5%.

### Selection of intervention and control clusters/villages

**Figure 2:**
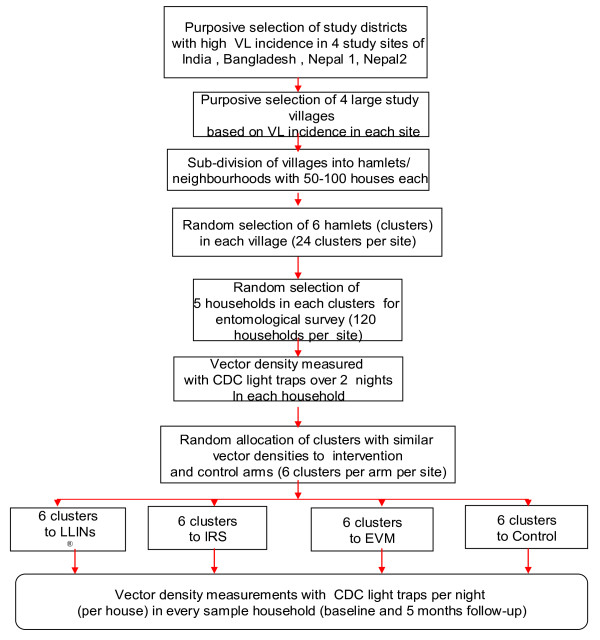
**Flow chart of study implementation in each site**.

In order to identify potential study sites with high vector densities, villages with a reportedly high VL endemicity (see the study areas section) were either selected from a list of villages with a high VL incidence (four out of 20 in Bangladesh and four out of 10 in Nepal (Tribhuvan University) or four villages with the highest VL incidence in the previous 3 years (in India and Nepal - BP Koraila Institute of Health Sciences; Figure 2). These large villages were subdivided into smaller units containing 50 - 100 houses each. Each of these sub-village units represents a cluster; six clusters were randomly selected giving a total of 24 clusters per study site. In all sites, the houses were numbered and informed consent was sought from the household heads.

### Selection of households for measuring sand fly densities

For the measurement of sand fly densities within each cluster, five houses at a minimum distance of 10 m were randomly selected by simple random sampling from a list of households after stratification into houses for humans only (HH) and mixed houses (MH) for humans and animals (no such stratification was carried out in Bangladesh where there were no MHs). With six clusters and five houses in each of the four arms, a total of 120 study houses were selected in each site (Figure 2). However, the interventions covered all the houses in each cluster.

### Cluster allocation to intervention arms

After the entomological baseline study, the 24 clusters in each site were placed into three groups of sand fly density - high, intermediate or low. Within each group, the clusters were randomly allocated to IRS, LLIN, EVM or control. In this way four arms were obtained, each containing six clusters (villages) but with a similar baseline vector density.

### Study arms and interventions

In the intervention clusters of the four study sites in the three countries the following measures were taken:

• IRS: in Bangladesh this was done using deltamethrin (K-Otrine 5%, Aventis Bayer company, target concentration 20 mg active ingredient per square metre); in India we used DDT 5% (target concentration 1 g/m^2^, Hindustan Insecticide Limited); and in Nepal we used alpha-cypermethrine (target concentration 0.025 gm/m^2^, Gharda Chemical LTD, Mumbai, India). IRS was carried out by the district vector staff, except for Bangladesh where the research team themselves had to do it in the absence of a vector control programme. Prior to the day of spraying, the head of household was informed of the procedure and date of IRS and was asked to sign a consent form. On the day of spraying, family members removed food, clothing, bed linen and animals before spraying. A spray field worker applied the insecticide to the interior (in Bangladesh also to the exterior) walls of the house and cattle sheds, up to 6 ft high, targeting the cracks and crevices. Quality control was done by the research team. Every household of the entire village/cluster was treated. Heads of household were told not to re-plaster their walls. The insecticides used in the study (as well as in routine vector control programmes) are efficacious against sand fly vectors (see the bioassay results below).

• LLIN: PermaNet^® ^nets (Vestergaard-Frandsen Company, Lausanne, Switzerland) with small mesh (156 holes/in^2^), polyester, resin coating containing deltamethrin (55 mg/m^2^) were distributed in all households of this study arm during the week 0 after the first survey in order to cover all household members based on reported sleeping arrangements. Two net sizes (160 × 180 × 150 cm and 100 × 180 × 150 cm) were used depending upon household members and sleeping arrangements.

• EVM: Trained community mobilizers met with each family to discuss the typical resting and breeding sites in and around houses and the appropriate ways to reducing them. In Nepal and India wall plastering with a lime/mud mixture was promoted, the lime being provided free of charge to the households. In Bangladesh plastering was done with mud only. The heads of household did the wall plastering themselves as it is a common practice. In Bangladesh a token incentive was provided to the children and housewives in order to encourage their participation. After the initial plastering activities, the community mobilizers conducted weekly home visits and meetings to promote the continuing filling of cracks and crevices in houses and cattle sheds [15].

• Control: Control clusters were similar in all ways to the intervention clusters; no specific vector control intervention was carried out.

### Entomological monitoring

Vector density in each cluster was monitored by CDC light traps [16] during two consecutive nights, pre-intervention (November 2006) and post-intervention (April 2007), in five randomly selected households in each intervention and control cluster (5 houses × 2 nights × 24 clusters = 240 measurements per site). Each study team completed the entomological survey within 3 - 4 weeks using 8 - 10 light traps at the same time. In each house they located one light trap between 6 pm and 6 am in a standardized way: in the corner of the main room 1-2 in from the wall and 6 in apart on the floor. The captured sand flies were examined on the day of collection. Test tubes were left in -20°C for 20 min, or chloroform-soaked cotton was used to kill the sand flies. The identification of sand flies was based on external morphological characteristics seen under a binocular microscope. The sand fly species was identified, sex and the abdominal condition of females were noted separately (*P. argentipes, P. papatasi, Sergentomyia *spp.) [17].

### Bio-assays

In order to determine the efficacy of the insecticide sprayed surface and LLINs, wild caught *P. argentipes *in Bangladesh, India and Nepal were exposed to randomly selected sprayed surfaces for 30 min and to randomly selected LLINs for 3 min using the World Health Organization (WHO) cone bio-assay method, including four replicates and one control in each assay [18]. The 24 hour mortality was higher than 80% in all study sites and will not be considered further in this paper.

### Statistical analysis

All analyses are based on total sand fly counts collected by CDC light traps; *P. argentipes*, the VL vector, represented 51% of all sand fly catches (range 48% - 59%). For the main efficacy analysis, data from the four study sites were pooled, including the baseline survey and follow-up survey, five months (for India and Nepal) and six months (Bangladesh) later to determine the medium term effect of the intervention. Additionally, the site-specific analyses were carried out. It was found that the Poisson distribution fitted the data and all analyses were done under that assumption.

Multilevel modelling with sample clusters (hamlet/neighborhood) as the second level of clustering was applied. The Poisson-regression procedure in STATA 10.1, with a robust sandwich estimator for clustering, was used in the analysis. Baseline data and post-intervention data were analysed both separately and in a longitudinal model. In the latter model an interaction term of being in the intervention arm at follow-up was included in order to estimate the effect of the intervention. The intervention effect was then estimated as the difference of the differences and should be zero if there is no intervention effect and negative if there is a larger reduction in the intervention clusters than in the control clusters:

Effect of intervention: (B-A)-(D-C): A = baseline value for the intervention group; B = post-intervention value for the intervention group; C = baseline value for the control group; D = post-intervention value for the control group

Technically the regression model has the following structure:

Count = Intercept + a*Treatment + b*Time + c*Interaction + error, where treatment is one if it is the intervention and zero if it is the control; where time is 1 if follow up is 5 months after intervention and zero if baseline; and where interaction is 1 if the intervention group at follow up. In the tables only the *c*-coefficient and its *P*-value are given.

Significances are stated at 5% level and 95% confidence intervals are given. In this paper we will focus on the pooled analysis of the four study sites in order to draw general conclusions but will also present site specific results.

The main outcome variable was 'total number of sand flies per trap per night' before and 5 months after intervention. The following variables were controlled for in some of the analyses: 'type of dwelling' (houses with only human inhabitants versus mixed dwellings with humans and cattle) and 'type of wall' (walls with bricks, concrete, mud versus bamboo sticks and other precarious materials).

## Results

In order to achieve a higher power of testing the comparative efficacy in reducing sand fly density through three interventions (IRS, LLINs, EVM) we undertook a pooled analysis of the results in the three countries. To capture the difference among the study sites, individual site specific analyses were done which was particularly important in order to understand the different effects of the various EVM interventions (lime plastering in India and Nepal; mud plastering in Bangladesh).

### Pooled analysis

Table 1 summarizes the vector densities at baseline and after intervention for all the study sites. The mean and confidence limits per intervention per survey, as well as the *P*-values from testing the difference in mean between the three interventions and the control at baseline and follow up survey, are presented. Vector densities at baseline indicate no or only small but non-significant differences. The effect of the interventions on vector density is shown in the follow up survey 5 months later. The intervention effect is very clear for IRS and less impressive, but significant, for EVM and LLIN compared to the control clusters where the vector density increased. Due to this increase, the net effect of the interventions can be better estimated in the following way: the estimated intervention effect in terms of reduction in sand fly counts in the simple model showed a 72.4% reduction for IRS, a 42.0% reduction for EVM and a 43.7% reduction for LLIN.

**Table 1:** Number of sand flies per house (trap) per night at all four sites pooled in Nepal, Bangladesh and India (Unadjusted mean counts of sand flies at baseline and at 5 months follow up with cross-sectional testing).

	**LLIN**			**IRS**			**EVM**			**Control**			***P*-value test of difference**		
		**CI 95%**			**CI 95%**			**CI 95%**			**CI 95%**		**LLIN versus Control**	**IRS versus Control**	**EVM versus Control**

Survey*	**Mean**	**Lower**	**Upper**	**Mean**	**Lower**	**Upper**	**Mean**	**Lower**	**Upper**	**Mean**	**Lower**	**Upper**			

Baseline	9.92	7.28	13.53	12.32	9.54	15.92	13.21	9.94	17.55	9.41	6.97	12.71	0.798	0.184	0.108

5 months follow-up	8.32	5.56	12.45	6.14	4.00	10.47	10.39	7.56	14.29	12.15	8.68	17.00	0.160	0.035	0.503

In Table 2 the *P*-values for testing the intervention effect are presented when controlling for differences in baseline values for the interventions and control, respectively.

**Table 2:** Longitudinal regression analysis of the pre-post control group design

		**LLIN**	**IRS**	**EVM**
**Model**	**Parameter**	**Coefficient (*P*-value)**	***P*-value**	***P*-value**

Simple (crude estimates)	Intervention effect†	-0.43 (0.042)	-0.95 (<0.001)	-0.49 (0.024)
	
	Reduction in counts (95% CI)‡	-4.34 (-8.57,-0.10) 43.7%	-8.92 (-13.20,-4.64) 72.4%	-5.55 (-10.57,-0.53) 42.0%

				

Full	Intervention effect	-0.42 (0.044)	-0.94 (0.001)	-0.49 (0.025)
	
	Type of wall	-0.02 (0.881)	-0.17 (0.260)	0.01 (0.925)
	
	Type of dwelling	0.00 (0.996)	0.27 (0.032)	0.26 (0.020)

Type of dwelling	Intervention effect	-0.43 (0.042)	-0.95 (<0.001)	-0.49 (0.024)
	
	Type of dwelling	-0.01 (0.970)	0.25 (0.043)	0.26 (0.021)

Type of wall	Intervention effect	-0.43 (0.044)	-0.94 (<0.001)	-0.49 (0.025)
	
	Type of wall	-0.02 (0.876)	-0.16 (0.289)	0.02 (0.875)

The intervention effect and covariates are tested in four different longitudinal models; simple not controlling for any covariates, full model controlling for type of wall and type of dwelling and the two semi controlled models. Intervention effect in terms of sandfly counts and percentage reduction is given for the simple model. P-values for the regression parameters are presented. Take into account the multiplicative structure of the regression model. (n = 840)

* (B-A)- (D-C), with A and B being baseline and 5 months sandfly count in the intervention group and C and D being baseline and 5 months sandfly count in the control group. Percent reduction = level of reduction (e.g. 4.34) divided by baseline intervention group count (e.g. 9.92) times 100 (43.7% in this example).

Note: The pooled analysis of EVM has to be interpreted with caution as two different EVM methods were applied: lime plastering in India and Nepal; mud plastering in Bangladesh (see site specific differences in the text)

†The intervention effect and covariates are tested in four different longitudinal models; simple not controlling for any covariates, full model controlling for type of wall and type of dwelling and the two semi controlled models. Intervention effect in terms of sand fly counts and percentage reduction is given for the simple model. P-values for the regression parameters are presented. Please observe the multiplicative structure of the regression model. (n = 840)

‡ Percentage reduction

LLIN, long-lasting insecticide treated nets; IRS, indoor residual spraying; EVM, environmental modification; CI, confidence interval

Controlling for type of dwelling (house for humans only or mixed houses with humans and cattle) and/or type of wall does not alter the significance of the intervention effects. Type of wall is insignificant for all arms, while type of dwelling is significant for IRS and EVM.

### Site specific analysis

While in all sites the same LLIN product was introduced, the chemicals for IRS were different (pyrethroids for Nepal and Bangladesh; DDT for India) but all of them had a high efficacy in the bioassays. EVM was done with different materials: lime plastering in India and Nepal and mud plastering in Bangladesh.

The estimated intervention effects for site specific analysis are given in Table 3 showing the following pattern:

**Table 3:** Site specific analysis.

**Site**	**LLIN†**	**IRS†**	**EVM†**
Nepal 1 (*n *= 120)	-3.80 (-14.7, 7.1) 22%	-10.7 (-26.7, 5.3) 53%	-10.6 (-22.3, 1.2) 51%

Nepal 2 (*n *= 240)	-2.11 (-8.4, 4.2) 16%	-8.5 (-16.0, -0.9) 52%	-0.6 (-10.7, 9.5) 4%

Bangladesh (*n *= 240)	-8.2 (-13.8, -2.7) 68%	-10.3 (-13.8, -6.8) 94%	-0.8 (-5.4, 3.8) 9%

India (*n *= 240)	-2.9 (-3.7, -2.1) 298%	-7.1 (-9.5, -4.7) 124%	-12.0 (-15.8, -8.1) 108%

Crude estimated reduction in counts without controlling for potential confounders. Reduction in counts (95% confidence interval).

*(B-A)- (D-C), with A and B being baseline and 5 months sandfly count in the intervention group and C and D being baseline and 5 months sandfly count in the control group.

† Percent reduction = level of reduction divided by baseline intervention group count. Larger than 100% when the intervention group has decreased while the control group has increased.

• IRS resulted in significant sand fly reductions in all sites independent of type of walls or dwelling or type of insecticide (DDT or pyrethroids).

• LLINs had a significant negative effect on sand fly densities in India and Bangladesh, but not in the two Nepal sites (see the discussion section)

• EVM using mud for wall plastering in Bangladesh was not effective; EVM using lime plastering significantly reduced the sand fly densities in India and Nepal (Sarlahi and Sunsari districts) but not in the other Nepal site (Morang district; see discussion)

## Discussion

### Entomological evidence regarding the efficacy of IRS, LLIN and EVM

The study has shown that IRS in particular, under quality controlled conditions and implemented by dedicated research teams, was efficacious in reducing the indoor sand fly density. LLINs and EVM were also able, under certain but not fully understood conditions, to reduce VL vector densities at least for 5 or 6 months after the intervention, independent of housing conditions and the presence or absence of cattle. The pooled results on intradomestic sand fly reduction (Table 1 and 2) were most likely due to the overall reduction of the sand fly population in each of the localities as the intervention was applied to all the houses of the selected clusters houses and not only the five houses where the measurement was taken. This approach is different from interventions directed only to individual houses as described by Dinesh *et al*. (2008) [16,19] in Bihar, where LLINs did not necessarily reduce the intradomestic VL vector densities but may have protected the occupiers of the selected houses from sand fly bites.

The site-specific data regarding LLINs and EVM have to be interpreted with caution because the limited number of clusters per arm provided uncertain estimates (wide confidence Intervals). LLINs had a significant effect on the number of sand flies in India and Bangladesh, but not in the two Nepali sites; when taking them together the intervention effect was also significant. This is confirmed by another VL vector intervention study using LLINs (unpublished, Kalanet Project). For the protective efficacy of LLINs, it has to be taken into consideration that CDC light trap captures monitor sand flies outside the bed nets, so that the actual protection for people sleeping under a LLIN from infective bites is most likely to be higher. The effect of LLIN on leishmania incidence, both cutaneous and visceral, has been shown in several studies in Latin America, Africa and the eastern Mediterranean region [20]) but not yet in the Indian subcontinent. Further research is necessary to establish the nature and extent of LLINs as protective tools against VL transmission and one ongoing community trial is addressing this question .

EVM using mud only for wall plastering does not seem to work. This confirms the results reported in an earlier case-control study in Bihar, India, where mud-plastered walls were even a risk factor for VL [21]. Lime plastering in India, and in one of the two Nepali sites, reduced sand fly density; the non-significant reduction in the other site probably being either due to chance or to the acid soil (which reduces the effect of lime) or the poor quality of the lime product. Although not a dramatic result, the effect of lime plastering confirmed the previous work by Kumar *et al*. [13] which showed that lime limits indoor sand fly breeding (calcium hydroxide when reacting with water is a toxic substance for sand fly larvae). However, the effect is limited to 6 - 7 months [20] and the cost factor is important: the annual unit costs per house served were calculated by Das *et al*. [22] and they included the costs of manpower, transportation and materials (lime, insecticides, LLINs, promotional materials). The unit costs were much lower for IRS (US $5.7; range 2.4-11.7) and LLINs (US $4.5; 3.5-5.1) than for EVM (US $8.8; 5.3-14.0).

### Study limitations

In addition to the above mentioned limitations resulting from the restricted sample sizes for site specific analyses, it should be reiterated that this study was conducted under fairly controlled conditions which are not easily applicable in a national vector control programme. However, it provides an indication of the way in which to undertake treatment programmes.

## Conclusion

When the above study results were discussed with the policy makers involved in the VL elimination initiative at two meetings in India and Nepal with the Regional Technical Advisory Group, a group of advisers (including programme managers, policy makers and academics from the three countries) agreed that the study results confirm that IRS should continue to be the main vector control strategy used in India (so far they have used DDT) and in Nepal (so far they have used pyrethroids) [23]. However, the efficacy, timing of spraying rounds and staff performance under the real conditions of a national programme should be assessed by the research teams, including the evaluation of insecticide resistance and subsequent monitoring through sentinel surveillance. A recent literature review has shown that insecticide resistance in the Indian subcontinent is not yet widespread but that high IRS coverage, frequency of sprays and choice of insecticide will be important factors in achieving an impact on the vector population and reducing the spread of resistance [20]. In Bangladesh, where vector control has been practically abandoned over the past decades but the use of bed nets is widespread (over 90% in the VL endemic districts according to a recent study [5], the net treatment with long-lasting pyrethroid formulations will probably have the most immediate effect in reducing the vector populations and man-vector contact. However, a cost-effective delivery strategy has yet to be developed and validated in a research context and under programme conditions.

Policy makers acknowledge that such implementation research can contribute crucial information for cost-effective strategies [24]. However, as new questions arise, researchers will have to work hand-in-hand with control managers to ensure correct interpretations and optimal use of data.

## Abbreviations

WHO: World Health Organization; VL: visceral leishmaniasis; IRS: indoor residual spraying; LLIN: long-lasting insecticide treated nets; EVM: environmental modification; DDT: dichlorodiphenyltrichloroethane; HH: houses containing only humans; MH: houses containing humans and animals.

## Competing interests

The authors declare that they have no competing interests.

## Authors' contributions

All authors contributed in to the design, implementation and analysis of the results. This is multicentric study and the article was prepared by the joint efforts of the authors.

## Pre-publication history

The pre-publication history for this paper can be accessed here:


